# Cell Based Treatment of Autoimmune Diseases in Children

**DOI:** 10.3389/fped.2022.855260

**Published:** 2022-05-09

**Authors:** Olcay Y. Jones, Deborah McCurdy

**Affiliations:** ^1^Division of Pediatric Rheumatology, Department of Pediatrics, Walter Reed National Military Medical Center, Bethesda, MD, United States; ^2^Division of Allergy, Immunology, and Rheumatology, Department of Pediatrics, University of California, Los Angeles, Los Angeles, CA, United States

**Keywords:** stem cells, mesenchymal, transplant, treatment, autoimmune, children

## Abstract

Mesenchymal stem cells have recently been recoined as medicinal signaling cells (MSC) for their ability to promote tissue homeostasis through immune modulation, angiogenesis and tropism. During the last 20 years, there has been a plethora of publications using MSC in adults and to lesser extent neonates on a variety of illnesses. In parts of the world, autologous and allogeneic MSCs have been purified and used to treat a range of autoimmune conditions, including graft versus host disease, Crohn’s disease, multiple sclerosis, refractory systemic lupus erythematosus and systemic sclerosis. Generally, these reports are not part of stringent clinical trials but are of note for good outcomes with minimal side effects. This review is to summarize the current state of the art in MSC therapy, with a brief discussion of cell preparation and safety, insights into mechanisms of action, and a review of published reports of MSC treatment of autoimmune diseases, toward the potential application of MSC in treatment of children with severe autoimmune diseases using multicenter clinical trials and treatment algorithms.

## Current Challenges in Pediatric Rheumatology

**FIGURE 1:**
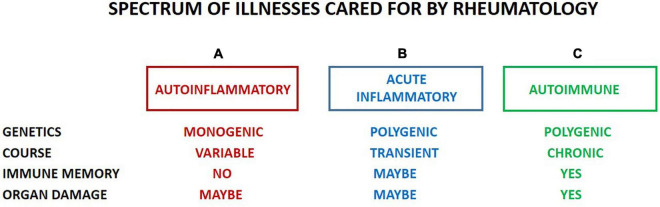
Pediatric rheumatology is involved in inflammatory conditions in children that range from monogenetic **(A)**, i.e., autoinflammatory syndromes to polygenic **(B,C)**. The latter can be one time occurrence (**B**; such as Kawasaki Disease or MIS-C) or chronic and long term **(C)**. Autoimmune conditions **(C)** can be systemic (i.e., affecting 2 or more target organs; such as SLE, JDMS, SSc) or single organ specific (such as Rheumatoid factor + RA, T1DM, autoimmune thyroiditis, uveitis, IBD and Multiple Sclerosis). We postulate that most conditions under B are triggered by infections and the determining factor between the two polygenic inflammatory conditions **(B,C)** is the presence or absence of an adversary immune memory. Should breakage of tolerance occur, there might be a progression from **(B,C)** (such as reactive arthritis to chronic arthritis) at varying speed and intensity based on host HLA, genetic risk factors, immune -repertoire and -memory, and the properties of the triggering event.

The subspecialty of pediatric rheumatology cares for children with autoimmune, autoinflammatory and immune dysregulatory illnesses that occur with an incidence of 1 in 10,000 to 1 in a few million children per year. Although the prevalence is not fully known, it is estimated that in the United States alone, 24 million people or over 5% of the population have an autoimmune disease and a proportion of the affected are children requiring care from a pediatric rheumatologist (NIH Autoimmune Diseases Coordinating Committee: Progress in Autoimmune Diseases Research, March 2005). Figure 1 shows the types of conditions cared for by the subspecialty; these include those confined to children, such as Juvenile Idiopathic Arthritis (JIA), and those that can affect a wide age range, such as systemic lupus erythematosus (SLE), dermatomyositis (JDMS), scleroderma (SSc), rheumatoid arthritis (RA), and inflammatory bowel disease (IBD). Within this group, children constitute about 10–20% of the total number representing the tail end of the bell-shaped curve for age of onset (1). Not only do children have longer time span to cope with the illness, but also the evidence suggests, on average, the severity of illness can be more pronounced (2, 3). This brings challenges to control disease activity and damage over time to ensure the child’s growth into a productive adulthood.

Current treatment modalities are designed primarily to provide immunomodulation without any direct support to *de novo* regeneration (4–7). Steroids are powerful to down regulate various inflammatory pathways, but prolonged usage is unacceptable for numerous adverse effects at young ages (8). In the last 20 years, targeted treatment using biological response modifiers have been successful steroid sparing agents particularly in arthritis, however, long term adverse effects of these medicines remain unknown. Successful treatment of systemic illnesses has been more limited; potent immune suppression to dampen immune memory requires combination therapy using steroids, chemotherapy, biologic response modifiers and recently Jak kinase inhibitors. Although, these agents bring increased treatment options, this is at the expense of escalated risk for serious infections and, yet unknown, adverse repercussions. While there has been significant progress on the treatment protocols for our patients, still, in long-term follow-ups, immune mediated inflammatory diseases (IMID) ranked among the top ten leading causes of death and emphasizes the high burden of inflammation (9).

## Cell Based Treatment for Autoimmune Diseases

Humankind is dependent on two kinds of multipotent progenitor cells throughout life (10) both are harbored in the bone marrow (BM): hematopoietic stem cells (HSC) and mesenchymal stem cells (MSC). The latter, recently recoined as Medicinal Signaling cells (11), are pericytes located over the abluminal surface of blood vessels, and found not only in BM, but also throughout tissues (12, 13). While HSC are the progenitors of blood cells (i.e., leukocytes, erythrocytes and platelets), MSC can differentiate in somatic cells including adipose tissue, chondrocytes, osteocytes and myocytes necessary for growth, regeneration, and tissue repair (14). In addition, MSC can modulate leukocytes to reduce inflammation and preserve tissue homeostasis by angiogenesis and tissue tropism (15). MSC can be expanded *ex vivo* into large numbers without senescence or malignant transformation. Morphologically they are adherent fibroblast-like cells with surface markers positive for CD105, CD73, and CD90 and negative for CD34, CD45- (16). Functionally, MSC are immunomodulatory through evolutionarily highly conserved paracrine factors [such as indoleamine 2,3-dioxygenase (IDO), prostaglandin E2 (PGE2), Heme Oxygenase-1 (HO-1), transforming growth factor β (TGF β)], as well as, by release of exosomes carrying compact cargo customized to the needs in microenvironment, and by cell to cell contact (17–19). As a result, there is down regulation of innate and specific immune system and upregulation of regulatory feedback loops as evidenced by increased M2 Macrophages, IL-10 and T regulatory cells (Treg).

It is important to note that information encoded within the HSC provides the blueprint for the composition of the immune repertoire and the set points of immune regulation toward environmental insults. This concept was proven experimentally in the 1980s by achieving cure upon myeloablative BMT of inbred lupus mice models using allogeneic donor cells from healthy strain (20, 21). Similar results were shown using myeloablative mixed chimerism protocols suggesting importance of immune regulatory networks (22, 23). Non-myeloablative mixed chimerism BMT was effective for survival (71.4%) and preserved kidney histopathology in treated lupus mice at 62-week follow-up, but it required co-transplant of MSC (24). Response to treatment with MSC alone varied based on lupus strain (25, 26). However, a comparative assessment of treatment response among different lupus strains to the same MSC protocol remains to be seen.

There are case reports confirming that transplant of allogeneic (related or unrelated donor) HSC transplant can lead to cure in certain autoimmune diseases. Most studies involved oncology patients with co-existing autoimmune disease who underwent BMT for cancer. Although, so far this is the only known treatment that can promote cure, it can be associated with over 20% mortality and high risk for acute or chronic graft versus host disease (GVHD) (27). In late 1990s, the concept of “setting the clock back” so as to eradicate immune memory by myeloablative autologous (patient’s own) BMT was envisioned (26). Over 1,500 patients with various autoimmune diseases were treated. The long-term outcomes of this approach is summarized in Tyndall (28) and Farge et al. (29): 100-day treatment related mortality (TRM) was 1% for RA and 11% for lupus patients. The 5-year survival was at 85%, remission rate was about 30%. About 5% of all treated were under 18 years old; among those, 65 were patients with JIA. During long-term follow-up of 34 children for up to 5 years, 53% achieved remission, 21% were resistant to treatment and 9% were deceased mostly from infection (30). There have been only a few studies on non-myeloablative transplant protocols (31–33); it is based on gentle conditioning; therefore, it can be a promising direction for reduced TRM on selected patients.

In analogy to HSC, it has been suggested that autoimmune diseases may in part be propagated by abnormal properties of MSC (34). There is limited evidence in support of this concept when *ex vivo* expanded MSC from patients with various autoimmune diseases are examined by a battery of tests including cell morphology, doubling time, signs of senescence, cell surface markers, and functional studies on immune modulation and angiogenesis. For instance, bone marrow derived MSC from patients with SLE (25, 35–37) showed evidence of distorted cell morphology, early senescence, and slow growth to confluence *in vitro* even though the surface markers and differentiation potential remain compatible to those from healthy controls. Functionally, the immunomodulatory activities may vary from normal (36) to impaired (35). Similar observations have been reported in scleroderma patients: when bone marrow derived MSC from scleroderma patients were cultured *in vitro*, the percentile of endothelial like MSC was significantly decreased, along with signs of early senescence and impaired capillary morphogenesis when compared with healthy controls (38–41). Interestingly, the senescence and immunomodulatory activities of MSC from SLE or scleroderma patients can be improved by inhibition of JAK-STAT or activation of mTOR pathways, respectively (42, 43). There have been similar observations that properties of MSC may (37) or may not (44) be altered in organ specific autoimmune diseases. The evidence so far does not suggest a prominent effect of iatrogenic influences on MSC that the patient may be exposed to Mancheño-Corvo et al. (45), but the literature in this area has been sparse and does not allow for full conclusions.

Nonetheless, these studies have encouraged applications of allogeneic MSC in clinical trials and paved the way for development of off the shelf products. In clinical trials discussed below, the MSC were prepared from adipose tissue, umbilical cord or bone marrow samples. Properties of MSC based on the source tissue is an ongoing area of research (46–48). Although surface phenotype remains similar, there are significant differences in gene expression profiles and differentiation potential based on the tissue of origin (49) even when they are derived from the same donor (50). The treatment outcomes, however, appear to be comparable irrespective of tissue of origin that correlates with the report that the immunomodulatory activities of MSC derived from different tissues of a single donor were reported comparable (51).

**FIGURE 2:**
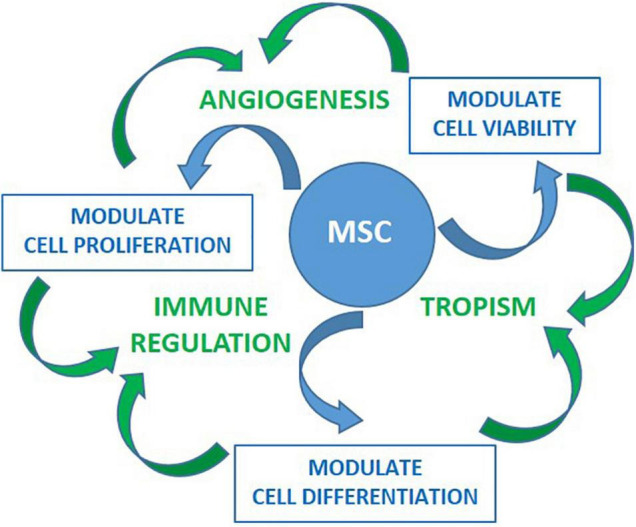
Preclinical studies have shown effects of MSC on cell viability, proliferation and differentiation toward preserving homeostasis. These are highly coordinated activities delivered through evolutionarily conserved mediators as well by cell-to-cell contact toward promoting angiogenesis, tropism and/or immune modulation. These activities are tailored based the signals received from the microenvironment such as those through proinflamatory cytokines and Danger- Associated Molecular Patterns (DAMPs) receptors. Once activated, MSC can modulate both innate and specific immune system *in vitro*. This is accomplished by inhibiting activation and proliferation of effector cells of myeloid (neutrophil, monocyte/macrophage, dendritic) and lymphoid (Th1, Th17, B, NK) lineages, as well as, by promoting differentiation and expansion of regulatory cells (Treg, M2 macrophages, and myeloid derived suppressor cells). *In vivo* immunomodulatory activities of MSC have been supported by the findings of increased Treg and decreased Th17 cells in several studies. Improvement of immune regulation coupled with tropism and angiogenesis is promising for MSC treatment allowing reparation of tissues damaged by inflammation.

Pro’s and Con’s of cell based treatment is summarized in Table 1. While HSC is a once in a life- time event, MSC has the potential to be developed as a “rescue measure” that can be repeated on as needed basis. It is important to point out that the success of HSC transplant is dependent on engraftment. This is not the case for MSC. So far, there is no proven method to promote engraftment of MSC (52, 53) and the efficacy of MSC is likely to be based on paracrine factors to modulate immune regulations, promote angiogenesis and support vitality of stromal cells to improve and/or sustain homeostasis as depicted in Figure 2.

**TABLE 1:** Cell based treatment of autoimmune diseases.

Cells	Donor	Pro’s	Con’s	.
HSC	Allogeneic	Cure	Requires conditioning	
			High risk for GVHD, Requires HLA match High TRM	
	Autologous	No risk for GVHD	Requires conditioning No need for HLA match May or may not be a cure	
MSC	Allogeneic	Do not require conditioning No need for HLA match No risk for GVHD So far, high safety profile	Not cure Expect transient improvement	
	Autologous	Patient’s own cells	Concern for genetic factors limiting efficacy	

## Important Considerations in Cellular Treatment Protocols Based on Historical Foundation

Since the early 2000s (54, 55), there have been numerous open label phase I/II studies on MSC involving over one thousand patients globally to assess safety and feasibility of MSC transplant. In review of the literature, studies vary for cell source, cell type, treatment protocol, disease selection, and patient selection.

The groundbreaking studies that led this trend were the results of MSC treatment for steroid resistant acute (aGVHD) (56) on a 9-year-old child with malignancy. Shortly after, the protocols used for aGVHD were adapted in trials on autoimmune diseases based on the justification that the pathogenesis of both overlaps for immune mediated microvascular damage (57).

Most protocols involved introducing single cell suspensions of *ex vivo* expanded MSC at early passages (< passage 6; i.e., cells are harvested at less than 6th generation of culture expansion) into a host. The subjects were allowed to continue current medications. There was no conditioning except a small group of patients received cyclophosphamide as noted below. The variables include the source of cells (autologous versus allogeneic), type of cells (bone marrow derived, umbilical cord derived, or adipose tissue derived stem cells), route of infusion (systemic by intravenous, or intra-arterial, versus local injection), dose of cells (usually 1–2 million/kg) and frequency of infusions (once or given intermittently every few days to months). IV has been the most commonly used route of MSC treatment. The majority (>95%) of donor cells are trapped in lung vasculature and become undetectable within 2–3 weeks post infusion (58). It is postulated that most are taken out by the host’s killer lymphocytes (59, 60).

Assessment of disease activity and treatment response has been, by and large, by clinical tools including validated disease activity measures, basic laboratories and imaging. Few studies included advanced testing on immune parameters. To our knowledge, there has not been a histopathologic investigation to correlate tissue changes with reported outcomes in humans.

In general, the patient selection has been targeted to those with moderate to severe disease activity who failed to respond or had limitations that did not allow them to continue on conventional treatment. Among those, some had established tissue damage and impending organ failure. The treatment protocols for autoimmune diseases, so far, involved adults at ages of 18 years and above. There have been only few patients at ages down to 16 years old who were included in cumulative results without separating the data by age groups (61). To our knowledge, we were the first to report the experience on MSC treatment in Pediatric Rheumatology (62).

## Safety Data for MSC Based Therapies

MSC therapy is tantalizing to consider in autoimmune diseases as patients are chronically ill and current therapies are not curative. Most treatment regimens have significant immunosuppression and often have adverse side effects. MSC is generally thought to be devoid of major side effects and based on a review of the literature, over a thousand patients have received MSC treatment world-wide. The early concerns regarding the possibility of under reporting of adverse effects is slowly dissipating with increasing cumulative data through global engagement.

In two systematic analyses, the safety of MSC therapy was explored. In the first, Lalu et al. (63) used the MEDLINE, EMBASE, and the Cochrane Central Register of Controlled Trials and reviewed 2,347 studies with 36 studies that met inclusion criteria. The primary outcome adverse events were grouped according to immediate events (acute infusional toxicity, for example: fever), organ system complications, infection, and longer-term adverse events (death, malignancy). There were 1,012 participants with diverse clinical conditions (ischemic stroke, Crohn’s disease, cardiomyopathy, myocardial infarction, GVHD, and healthy volunteers). Eight studies were randomized control trials (RCTs) with 321 participants. Meta-analysis of the RCTs did not detect an association between acute infusional toxicity, organ system complications, infection, death, or malignancy. The major significant association with MSC therapy was a transient fever. Based on these reviews, the authors concluded that MSC therapy appeared to be safe, but more studies were needed. In another systematic analysis, Can et al. reviewed 93 peer-reviewed full-text articles and abstracts published by August 2017 that investigated the safety, efficacy and feasibility of UC- MSCs in 2,001 patients with 53 distinct pathologies. All studies noted therapeutic benefit and there were no long-term adverse events or tumor formation (55).

A retrospective study in a cohort of 404 patients with different autoimmune diseases who received MSC transplants from 2007 to 2016 was done in Nanjing University Medical School (64). Their endpoint was to evaluate the frequency of adverse events by using the Common Terminology Criteria for Adverse Events version 4.0 (CTCAE v4.0) (65). Based on this grading system, five grades were defined as: grade 1, mild: asymptomatic or mild symptoms, clinical or diagnostic observations only, intervention not indicated; grade 2, moderate: local or non-invasive intervention indicated; grade 3, severe or medically significant but not immediately life-threatening, hospitalization or prolongation of hospitalization indicated; grade 4, life-threatening consequences, urgent intervention indicated; and grade 5, death related to AEs. In this system, grades 3–5 are considered serious. Hyperacute adverse events were defined as occurring during and immediately after the infusion, while acute adverse events were defined as occurring from the second day to the first month after infusion. After the first month, subsequent infections and malignancies were analyzed. There were 11.9% of patient with hyperacute adverse events that included fever, headache, palpitation, facial redness, insomnia, and stomach discomfort, but all were classified as grade 1–2; mild. Patients with polymyositis or dermatomyositis (6/30 or 20%) or those over 40 years old (25/182 or 13.7%) had proportionately more hyperacute adverse events, but the numbers were small. Acute adverse events occurred in 4% in the first month after transplant including fever, hair loss, peeling skin, facial rash and cervical lymphadenopathy, that were mild, but there were 6 patients with infection, two with encephalorrhagia, and one cirrhosis with bleeding from esophageal varices resulting in 5 deaths. In this cohort, there were 45 deaths that occurred an average of 29.6 months after the MSC infusions. After 1 month, there were no cardiac, gastrointestinal, renal, pulmonary, neurological, or hematological adverse events. Death occurred in 45 patients, with 64.4% developing 3 years after MSC infusion. Infections remained a major concern, with 26.7% developing an infection. The most common cause of death was disease relapse (62.2%). Cancer occurred in 6.7% of patients. Again, those with dermatomyositis and polymyositis had proportionately the highest mortality. In this study, 26 patients were children (<18 yo). At the time of the report, 24 of the children had good outcomes during the 4–5 years following MSC transplantation. Two died from disease complications more than 100 days after the MSC transplant. The authors conclude that MSC therapy in autoimmune disease is safe and shows efficacy and concluded that the incidences of adverse events was acceptable to warrant MSC therapy in patients with autoimmune disease.

After infusion, the majority of the MSC are found in the lungs and the MSC are viable for about 24 h. At 24 h the MSC were also found in the liver. After 24 h, there are no viable MSC noted (66). The consequences of the MSC in the lung is not well known, but in patients with lung and cardiac disease there is concern that this massive influx of cells could result in activation of the cytokine and complement system. In patients with pulmonary hypertension, activation of the vascular system could result in acute ischemia that may be difficult to reverse. Although pulmonary embolism is a concern as a treatment related adverse effect, so far it is rarely reported. To this point, two recent studies on MSC treatment on severe COVID-19 infection did not report TRM (67, 68).

One of the later sequelae of MSC transfusion is the risk of teratoma formation, undifferentiated proliferation, or malignancy. In one clinical trial of MSC for treatment of advanced neovascular age-related macular degeneration, a MSC from a patient was found to have a mutation and the trial was stopped. It is not clear if this mutation was pre-existing or occurred during cellular preparation and re-programming (69). Further studies are needed for optimal preparation of MSC infusions and long-term data collection essential to determine the long-term risks of MSC. Although, the risk for interstitial lung disease or accelerated fibrosis initially was a great concern - for the potential of trapped MSC within the lung to differentiate into fibroblasts -, this, so far, has not been a reported adverse effect (70). Further studies are needed for optimal preparation of MSC infusions and long-term data collection essential to determine the long-term risks of MSC.

## MSC Treatment of Autoimmune Diseases—Overview of Publish Data

In the last two decades, there has been considerable data accumulated on applications of MSC for various autoimmune diseases. This is through the pioneering work mostly by the investigators of Far East and Middle East in single center-based trials at various academic institutions (64, 71–73). The major rheumatological illnesses studied, so far, include SLE, SSC, and Rheumatoid Arthritis (RA). There are only a few reports of use of MSC in dermatomyositis and vasculitis. There are also learning points from the experiences on other organ specific autoimmune diseases (MS, IBD, and DM) as well as from applications of MSC on intractable conditions of the newborns.

Lupus has been one of the most extensively studied disease models. In 2009, Dr. Sun and colleagues reported the first groundbreaking pilot study on four patients treated with allogeneic bone marrow derived MSC who achieved clinical and serological improvement during 12–18 month follow-up period (25). Similar disease control was achieved when umbilical cord derived MSC were used in some (74) but not all trials (75). The selection criteria included ongoing active lupus activity with SLE disease activity index (SLEDAI) score ≥ 8, inadequate disease control with high dose steroids (>20 mg/day) along with at least 6 monthly treatment of intravenous cyclophosphamide or at least 3 months of oral mycophenolate mofetil, refractory immune-mediated thrombocytopenia and refractory lupus nephritis (WHO IV/V with proteinuria ≥1,000 mg/24 h, serum creatinine ≥ 1.5 mg/dl or decreased creatinine clearance without end-stage renal failure. The MSC harvested from bone marrow or umbilical cord, expanded *in vitro* in media with fetal bovine serum. Cells from passage 3 to 5 were infused to patients intravenously at 1 × 10^6^/kg body weight. The outcomes were similar when MSC was given once weekly for in two intervals (76). Recently, the same center has published cumulative experience on 81 lupus patients for long-term outcomes (77). The treatment involved a total of 104 MSC infusions using bone marrow derived (22/81) or umbilical cord derived (59/81) MSC given intravenously once (66/81), twice (11/81), or up to 4 (4/81) times during the follow-up. In addition, 39/81 also received IV cyclophosphamide at 10 mg/kg/day × 3 just prior to MSC. The cohort was composed of moderate to severe, SLE who were resistant to various treatments (including cyclophosphamide in 59 out of 81 patients) prior to enrollment. Overall, the MSC treatment was safe and effective. Five-year survival was 68/81 and 37/81 achieved remission. Out of 37, 22 had complete remission (4 off treatment), 6 had partial remission and 9 relapsed. Fifteen out of 81 patients died from various non-treatment related events, 8 occurred within the first 12 months post MSC: 4 out of 8 of the deceased had pulmonary infection and 2 had cardiac compromise. Four of remaining 7 deceased at 31–83 months post MSC had continuing disease progression and ESRD. During the follow-up, majority of adverse events were centered on infections while 51 subjects remained on varying extents of immunotherapy. Laboratory parameters showed significant improvement in proteinuria and cytopenia, serum albumin and complement levels. Initial reports included decreased titers of double stranded DNA antibody, as well as increased blood T-regulatory (Treg) cells (CD4 + CD25 + Foxp3 +)—and decreased Th17- populations in the peripheral blood samples (73, 78). A follow-up case report on two lupus patients treated with autologous MSC also had increased Treg cells, but the clinical improvement was marginal (79). Increased levels of Treg post MSC is a reproducible finding, but further investigations are warranted to explore time course, and sustainability of blood lymphocyte profiles post treatment for its impact on treatment outcomes.

There is significant interest in MSC treatment of SSC for the paucity of effective treatment options, as well as, for the pathogenesis of the illness that is tightly coupled with the progeny of MSC, i.e., fibroblasts and endothelial cells (70). Initial results were encouraging on a small case series of four patients with leukemia who developed sclerodermatous chronic GVHD after bone marrow transplant (80). These patients improved after treatment with unrelated allogeneic bone marrow derived MSC injected *via* intra-osseous route. Follow up labs were significant for increased ratio of peripheral blood Th1 to Th2 cells. Keyszer et al. reported (81) their experience on five patients with severe and life-threatening SSC with positive Scl70 (*n* = 4) or positive anti-RNP (*n* = 1) autoantibodies. All 5 patients received a single intravenous infusion of related bone marrow derived MSC (0.2–1.8 × 10^6^/kg body weight). There was no treatment related mortality following shortly after infusion. The beneficial effect was observed mostly on skin findings; starting at 3 months post treatment, there was improvement of skin score for thickness and healing of ischemic ulcers. Two patients with cardiac involvement died at 6 and 23 months post MSC. Two patients with lung disease progressed- one requiring lung transplant. A case report from Italy (82) observed significant improvement of gangrenous ischemic ulcers after 3 monthly intravenous infusions of autologous bone marrow derived MSC (almost 1 × 10^6^/kg body weight/dose). There have been trials involving local injections of MSC in patients with scleroderma: A recent report from Japan (83) on 40 patients with peripheral arterial disease (11 with SSC and 29 with arteriosclerosis obliterans) reported improvement of ischemic ulcers based on a protocol involving surgical debridement followed by local intramuscular injections of autologous bone marrow derived stem cells (0.4–5 × 10^10^ total) and finally skin grafting to cover the open ulcers. At the 2-year follow-up, non-treatment related mortality rate, and recurrence rates were 27 and 18%, respectively. Nine percent progressed to require limb amputation. For treatment of childhood onset limited sclerosis, Scuderi et al. (84), injected autologous adipose tissue derived stem cells mixed with hyaluronic acid solution (8 × 10^5^/ml up to 10 ml) locally at the affected areas of skin in 6 patients (including one with generalized morphea, and one with En Coup De Sabre). There were no adverse effects. One patient had moderate and 4 had considerable levels of improvement at 1-year follow-up.

Treatment of refractory Rheumatoid Arthritis with MSC has been a global interest and a platform for industry sponsored trials using off the shelf MSC in the pipeline. Trials on arthritis started after a pilot study in Korea on 4 patients receiving autologous adipose derived MSC IV ± IA with a combined dose up to 500 × 10^6^/patient. The treatment was tolerated well without TRM. So far, there are over 400 patients treated with single or up to 3 weekly doses of allogeneic MSC at doses of ranging from 1 to100 × 10^6^ IV per infusion. Follow-up was 1–36 months (median 12 months), among 8 trials (85). A recent clinical trial from China (86) reported observations on 172 patients with RA who had history of partial response to conventional treatment. There were two study arms (1:1), one, with umbilical cord derived MSC (4 × 10^7^ × 1) and two, with cell free culture supernatant of MSC cultures. All subjects continued on DMARDS. There was significant improvement in the first arm, but not in the second arm. Furthermore, the improvement correlated with decreased serum proinflammatory cytokines (TNFα, IL6) as well as increased Treg that lasted for 3–6 months. There were no serious adverse reactions or treatment related mortality. Long-term follow-up of the same cohort was reported on 64 subjects (including 3 juvenile onset arthritis and 4 ankylosing spondylitis) 36 months post MSC treatment (61). The disease activity score (DAS28, HAQ), autoantibody titers for RF and cyclic citrullinated peptide antibody (anti- CCP) as well as blood inflammation markers (ESR, CRP) showed steady and significant decline over the 3 years. CBC, serum total immunoglobulins, liver and renal functions remained normal. Treatment of ankylosing spondylitis (AS) with MSC infusions also were safe and effective. In a study 31 patients with treatment resistant AS were treated with 4 weekly IV infusions of BM derived allogeneic MSC at 1 × 10^6^/kg/dose. There was no TRM or serious adverse effects. The clinical improvement correlated with MRI improvement at 20 weeks post treatment (87).

## Conclusion and Next Steps

Cell based treatment with HSC has cured many diseases in the last 5 decades (88) when there is no other remedy for illnesses like cancer or immune deficiency. Adaptation of this modality to autoimmune diseases, however, is challenging for TRM or GVHD. Even with autologous protocols, the conditioning regimens are concerning for high risk of infection. Adaptation of non-myeloablative protocols can be promising particularly for young children with known genetic risk factors and poor prognosis. This will require available full match donor and cross-disciplinary teams.

Recently, MSC treatment has been promising for multifaceted medicinal properties as it offers not only immune tolerance, but also vascular and somatic wellness (89, 90). This is important particularly for autoimmune diseases as long-term outcomes are determined by the balance between immune mediated damage and tissue regeneration. Unlike treatment with HSC where transplanted cells result with a binary outcome, i.e., all or none, MSC treatment should be considered as a transient, but personalized, therapy, and not a cure.

Treatment paradigms in complex diseases, particularly in rheumatology, are a moving target that is reconfigured along with advances in predictive biomarkers, preventive measures and targeted treatments. In the last 25 years, there has been a transition from an upright to a downward pyramid (91). We suggest MSC has the potential to become a component of a new treatment paradigm particularly for early intervention. While currently cell-based treatment is considered for life threatening conditions, this may change in time as the comfort level of using this therapeutic modality improves once further evidence becomes available on safety and efficacy. MSC may work better in early phases of the illness before permanent tissue changes develop. It is likely that some, if not most, of the patients treated with MSC will continue to require immunosuppressive regimens, albeit to a lesser extent, to prevent organ damage. MSC is not a treatment that can bring vitality back once there is effacement of tissue architecture and loss of tissue specific progenitor cells. Currently there is no effective protocol to accomplish engraftment of donor MSC and repair late-stage tissue damage *de novo*.

It is worth pointing out that, as a natural result of aging, it is well known that there is a decline in the numbers as well as the telomere length of MSC (92). This process is accelerated in patients with chronic inflammation. The mechanisms involved are not fully known but epigenetic changes are likely to be important. Advantage of introducing MSC may include sustained homeostasis through supporting *in situ* pericyte populations. Knowledge gain in reference biomarkers to assess *in vivo* landscapes for tropism and regeneration are important for successful applications of MSC and cell-free products of MSC (including exosomes) in the near future. In line with this concept, as the children have escalated regenerative capacity (7, 30) with ongoing natural physiology of growth, they may benefit from MSC more robustly when compared with adults.

**FIGURE 3:**
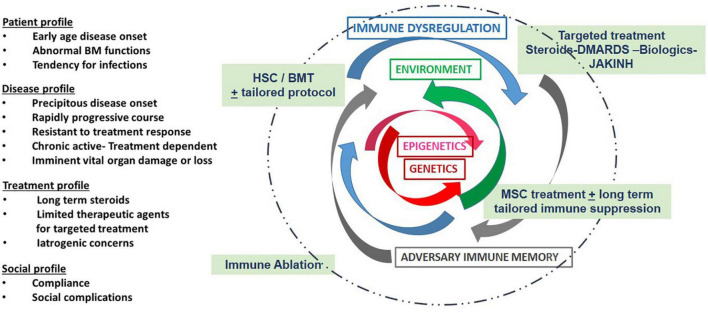
Depicts some of the considerations and challenges with MSC treatment: Treatment regimens are usually based clinical considerations as listed in the first column, i.e., genetic influences and disease course as well as the history of treatment response and social concerns. Genetic and epigenetic factors (red arrows) and their environment (green arrows) are unique to each patient. These combined factors may result in immune dysregulation (blue arrows) and a subsequent adversary immune memory (gray arrows). Therapies (in green text box) are designed to dampen inflammation. In selected patients, personalized cell based treatment protocols can be introduced to improve long-term outcomes and minimize damage. New algorithms are needed based on data derived from expanded laboratory panels and real time assessment tools that are now available through adaptations of existing technology and essential for patient centered care.

Currently there is no protocol tailored for pediatric autoimmune patients (Figure 3). Although, the 1st case reports for successful application of hematopoietic stem cells (HSC), as well as MSC treatment in medicine were on children for treatment of immune deficiency and malignancy, in 1968 (93) and 2004 (57), respectively. With the advancements of cellular therapy, commercial MSC products have been licensed for the indication of for pediatric steroid refractory GVHD in a number of countries including Japan, Canada, and New Zealand (94). On an important note, there has been significant progress on applications of MSC on intractable newborn diseases. In neonates, there are two recent review articles that site benefits of MSC in case reports or small trials in certain neonatal diseases including severe intraventricular hemorrhage (IVH), bronchopulmonary dysplasia (BPD), and necrotizing enterocolitis (NEC). The reports are encouraging in that there are no adverse events, the studies are small, but promising (95, 96). Most of these studies note the need for multiple infusions. There are now five phase I clinical trials in neonatal patients: 3 in BPD, 1 in IVH, and I in hypoxic ischemic encephalopathy (HIE) (96).

As the public becomes informed *via* the internet, patients and parents demand explanations and consideration of potential treatments. In the reported case of the three pediatric patients (1 with SLE, 1 with mixed connective tissue disease (MCTD) and one with JIA) who received MSC transplants, all were parent or patient initiated with great cost to the family (62). All reported improvement, but this may have been influenced by the difficulties and financial burden entailed to get the MSC transplant. Two of the patients had to travel outside of the US to receive the MSC transplant. As more information is available, patients and their families may seek this therapy, which on the internet has promoted as curative in some cases and benign. As pediatric rheumatologists, we strongly believe, it is for the benefit of our patients to bring awareness of this therapeutic modality and actively engage in its research to determine -first hand- its promise and, equally importantly, its potential adverse and long-term effects.

We do believe coordinated and multilateral initiatives involving academics, government, industry and patient advocacy groups are key for fast-track progress on multi-center and patient centered research to push the limits to reach cure.

## Author Contributions

OYJ and DM envisioned, crafted, wrote, and edited the manuscript. Both authors contributed to the article and approved the submitted version.

## Author Disclaimer

The views expressed in this article are those of the author and do not reflect the official policy of the Department of Army/Navy/Air Force, Department of Defense, or U.S. Government.

## Conflict of Interest

The authors declare that the research was conducted in the absence of any commercial or financial relationships that could be construed as a potential conflict of interest.

## Publisher’s Note

All claims expressed in this article are solely those of the authors and do not necessarily represent those of their affiliated organizations, or those of the publisher, the editors and the reviewers. Any product that may be evaluated in this article, or claim that may be made by its manufacturer, is not guaranteed or endorsed by the publisher.
